# Controlled-release hydromorphone and risk of infection in adults: a systematic review

**DOI:** 10.1186/s12954-023-00788-9

**Published:** 2023-04-28

**Authors:** Andrea C. Tricco, Amanda Parker, Areej Hezam, Vera Nincic, Fatemeh Yazdi, Yonda Lai, Charmalee Harris, Zachary Bouck, Ahmed M. Bayoumi, Sharon E. Straus

**Affiliations:** 1 Li Ka Shing Knowledge Institute, St. Michael’s Hospital, Unity Health Toronto, 209 Victoria St, Toronto, ON M5B 1T8 Canada; 2 Epidemiology Division Dalla Lana School of Public Health, University of Toronto, 155 College St Room 500, Toronto, ON M5T 3M7 Canada; 3 Institute for Health Policy, Management, and Evaluation, Dalla Lana School of Public Health, University of Toronto, 155 College St Room 425, Toronto, ON M5T 3M7 Canada; 4 Queen’s Collaboration for Health Care Quality Joanna Briggs Institute Centre of Excellence, School of Nursing, Queen’s University, 99 University Ave, Kingston, ON K7L 3N6 Canada

**Keywords:** Controlled-release hydromorphone, Knowledge synthesis, Systematic review, Risk factor, HCV, HIV, Infective endocarditis

## Abstract

**Background:**

Preliminary evidence suggests that people who inject drugs (PWID) may be at an increased risk of developing infective endocarditis (IE), hepatitis C virus (HCV) infection, and/or human immunodeficiency virus (HIV) infection from hydromorphone controlled-release formulation. The hypothesized mechanism is related to insolubility of the drug, which promotes reuse, leading to contamination of injecting equipment. However, this relationship has not been confirmed. We aimed to conduct a systematic review including adult PWID exposed to controlled-release hydromorphone and the risk of acquiring IE, HCV, and HIV.

**Methods:**

We searched MEDLINE, EMBASE, and Evidence Based Medicine reviews from inception until September 2021. Following pilot testing, two reviewers conducted all screening of citations and full-text articles, as well as abstracted data, and appraised risk of bias using the Newcastle–Ottawa scale and Effective Practice and Organization of Care tool. Equity issues were examined using the PROGRESS-PLUS framework. Discrepancies were resolved consistently by a third reviewer. Meta-analysis was not feasible due to heterogeneity across the studies.

**Results:**

After screening 3,231 citations from electronic databases, 722 citations from unpublished sources/reference scanning, and 626 full-text articles, five studies were included. Five were cohort studies, and one was a case–control study. The risk of bias varied across the studies. Two studies reported on gender, as well as other PROGRESS-PLUS criteria (race, housing, and employment). Three studies focused specifically on the controlled-release formulation of hydromorphone, whereas two studies focused on all formulations of hydromorphone. One retrospective cohort study found an association between controlled-release hydromorphone and IE, whereas a case–control study found no evidence of an association. One retrospective cohort study found an association between the number of hydromorphone controlled-release prescriptions and prevalence of HCV. None of the studies specifically reported on associations with HIV.

**Discussion:**

Very few studies have examined the risk of IE, HCV, and HIV infection after exposure to controlled-release hydromorphone. Very low-quality and scant evidence suggests uncertainty around the risks of blood-borne infections, such as HCV and IE to PWID using this medication.

**Supplementary Information:**

The online version contains supplementary material available at 10.1186/s12954-023-00788-9.

## Introduction

Hydromorphone is a prescribed semi-synthetic opioid recommended as second-line therapy for mild-to-moderate non-malignant pain and first-line therapy for severe non-malignant pain [1]. Hydromorphone is also a pain relief treatment option for cancer-related pain in the World Health Organization (WHO) updated guidelines [2]. Hydromorphone is available as immediate- and controlled-release formulations, and can be administered orally, intravenously, subcutaneously, through epidural or intrathecal, and intramuscularly [3].

People who inject drugs (PWID) are at high risk of hepatitis C virus (HCV) infection [4] and the WHO has set the target to reduce HCV prevalence up to 90% by 2035. There has also been a marked increase in infective endocarditis (IE) amongst PWID over the last decade [5–11]. These trends have mirrored the growing opioid crisis in recent years [12].

It has been suggested that the increasing availability of controlled-release formulation of hydromorphone might be associated with contributing to higher rates of these infections HCV, IE, and HIV due to contamination and reuse of injection drug preparation equipment (IDPE). Some authors have hypothesized that injection of controlled-release hydromorphone elevates the risk of blood-borne infections through multiple mechanism. First, controlled-release hydromorphone does not readily dissolve in water [13], leading to more frequent injections; injection risk will increase if injections are performed under non-sterile conditions. Second, because controlled-release hydromorphone is insoluble, it leaves behind a residue that may be kept for reuse [14] and/or shared with others. Bacterial or viral contamination of the residue or the equipment that stores the residue (i.e. the “cooker”) could contribute to the risk of blood-borne infection, even without sharing of needles or syringes. Third, laboratory data indicate that excipients within controlled-release hydromorphone may promote viral survival and infectiousness. However, the hypothesized association between injection of controlled-release hydromorphone and HCV, IE, and HIV infection amongst PWID has not been confirmed [15].

As such, we conducted a systematic review to examine the risk of IE, HCV, or HIV in individuals exposed to controlled-release hydromorphone compared with other opioids, as well as to determine the characteristics of individuals exposed to controlled-release hydromorphone experiencing these infections. Our specific research questions were: (1) “What are the rates of IE, HCV infection, and/or HIV infection in adults exposed to controlled-release hydromorphone compared with the rates of the same infections in people using immediate-release (oral) hydromorphone, injectable hydromorphone, and other controlled-release products globally?” and (2) “What are the characteristics of adults exposed to controlled-release hydromorphone who experienced IE, HCV infection and/or HIV infection, including previous treatment or hospitalization for opioid-related harms?”.

## Methods

### Protocol

We were commissioned by Health Canada to conduct this systematic review through the Canadian Institutes of Health Research Drug Safety and Effectiveness Network [16]. A preliminary review was conducted by Health Canada [17], which formed the basis for this systematic review. Health Canada was consulted at every stage of the systematic review process. A protocol was developed using the Preferred Reporting Items for Systematic Reviews and Meta-Analysis for Protocols (PRISMA-P) [18] and registered with the PROSPERO database (CRD42021289020). The systematic review methods were guided by the Cochrane Handbook [19] and reporting using the PRISMA 2020 Statement [20].

### Literature search

The literature search was developed by an experienced librarian (Dr. McGowan) and peer-reviewed by another librarian (Ms. Rader) using the Peer Review of Electronic Search Strategies (PRESS) checklist [21]. The electronic databases MEDLINE, EMBASE, and Evidence Based Medicine reviews were searched from inception until 20th September 2021. The literature search strategies for all databases can be found in Additional file 1: Appendix 1. Unpublished and difficult-to-locate information (i.e. grey literature) studies were searched using the Canadian Agency for Drugs and Technologies in Health (CADTH)’s Grey Matters guidance [22]. Searched Grey literature sources included various organizational websites, such as CADTH, CenterWatch, and the Canadian Medical Association Infobase. A full list of the grey literature sources is in Additional file 1: Appendix 2. Conference abstracts and dissertations identified through our literature search were screened for eligibility and attempts were be made to locate corresponding publications. Literature saturation was ensured by searching the reference lists of all included studies and related reviews.

### Eligibility criteria

The population of interest included adults aged 18 years or older. The exposure of interest was controlled-release hydromorphone (HCR) intake through any means or mode of administration, prescription, or illicit use. The comparators were immediate-release (oral) hydromorphone, injectable hydromorphone, or exposure to other opioids. The outcomes were incident cases of HCV, IE, and HIV. Only studies with a valid comparator were considered relevant, including randomized controlled trials, quasi-randomized trials, non-randomized trials, controlled before and after studies, interrupted time series, cohort studies, and case–control studies. No restrictions were applied based on study year, language of dissemination, or study duration.

### Study selection

A screening form (presented in Additional file 1: Appendix 3) was developed based on the eligibility criteria, and the team completed a training exercise using 50 citations to ensure adequate agreement was achieved. After completing two training exercises (achieving 40% and 70% agreement, respectively) and then revising our screening criteria form for clarity, all remaining titles and abstracts identified in the search were screened independently by pairs of reviewers (AP, AH, VN, DN, FY, YL, CH). All discrepancies were consistently resolved by a third reviewer.

Similarly, a training exercise was completed for screening of full-text articles, as seen in Additional file 1: Appendix 4, using 20 articles. After completing one training exercise (achieving 100% agreement), full-text articles were assigned to independent pairs of reviewers, and any discrepancies were consistently resolved by a third reviewer.

### Data abstraction

A data abstraction form (presented in Additional file 1: Appendix 5) was drafted to capture data on study characteristics, population characteristics, intervention details, and outcomes of interest. To capture data relevant to equity, the PROGRESS-PLUS criteria were used [23]. Relevant outcomes included incident cases of IE, HCV, or HIV. Due to the small number of included studies, a training pilot was not completed. Full data abstraction was completed by an independent pair of reviewers with discrepancies resolved by a third reviewer.

### Risk of bias assessment

The risk of bias appraisal was completed at the outcome level and was carried out by two reviewers independently using the Newcastle–Ottawa scale [24] and the Effective Practice and Organization of Care (EPOC) risk of bias tool [25]. Discrepancies were resolved by a third reviewer. Due to the small number of included studies and expertise on the team, a training pilot was not completed.

### Analysis and presentation of results

The review findings were summarized descriptively using summary tables. A random effects meta-analysis was deemed to be inappropriate for this review, due to heterogeneity observed across the limited number of studies.

## Results

### Literature search results

**Fig. 1:**
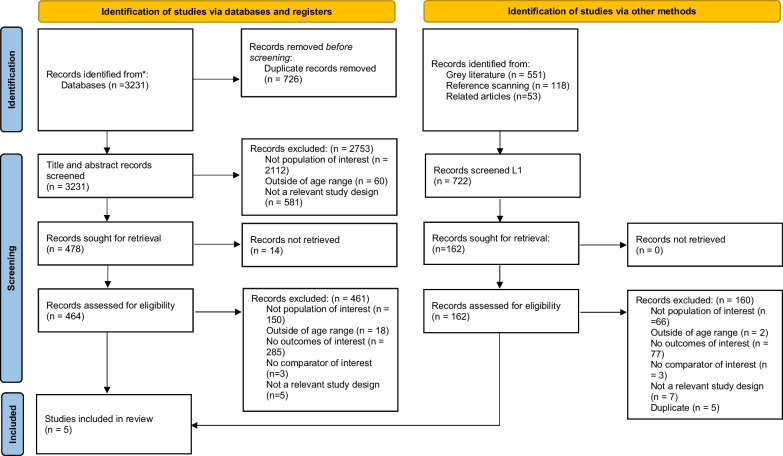
PRISMA flow diagram. From: Page MJ, McKenzie JE, Bossuyt PM, Boutron I, Hoffmann TC, Mulrow CD, et al. The PRISMA 2020 statement: an updated guideline for reporting systematic reviews. BMJ 2021;372:n71. doi: 10.1136/bmj.n71. For more information, visit: http://www.prisma-statement.org/

After screening 3,231 citations from the electronic database searches and 551 from grey literature searches, as well as 626 full-text articles, five studies fulfilled the eligibility criteria and were included (Fig. 1) [26–30]. A list of studies that were closely related to the inclusion criteria but eventually excluded is provided in Additional file 1: Appendix 6.

### Study characteristics

Four studies were cohort studies, and one was a case–control study (Additional file 1: Appendix 7). The studies were published in the years 2020 and 2021. All studies were conducted in Canada. The study duration was less than one year in two studies, less than five years in two studies, and more than five years in one study. The setting was multi-site in four studies and single site in one study. All studies examined injection drug use; no studies were found on other routes of administration for hydromorphone.

### Patient characteristics

The total number of patients were 4,208 across the studies (Table 1). The median number of patients was 196 across the studies, ranging from 26 to 3,790 patients (Additional file 1: Appendix 8). The average proportion of participants who were female per study was 48.4%. The most common comorbidity reported was alcohol use disorder (50%), yet comorbidities were not reported in nearly 50% of the studies.

**Table 1:** Summary of study and patient characteristics

Characteristics	Number (%)
Study characteristics (*n* = 7)	
*Year of publication*	
2020	4 (80%)
2021	1 (20%)
*Geographical region*	
Canada	5 (100%)
*Study design*	
Cohort	4 (80%)
Case control	1 (20%)
*Study duration*	
≤ 1 year	2 (40%)
≤ 5 years	2 (40%)
> 5 years	1 (20%)
*Setting*	
Multi-site	4 (80%)
Single site	1 (20%)
Patient characteristics	
Total # patients	4,208
Mean number of patients (range)	4,015 (26–3,790)
Mean % female patients (range)	48.425 (42.5–54)
*Age (mean/median)*	
≤ 40 years	1 (20%)
> 40 years	1 (20%)
Not reported	3 (60%)
*Studies reporting on outcomes*^*a*^	
Infective endocarditis	1
HCV	4
HIV	2
*Comorbidities*^*a*^	
Alcohol use disorder	3
Stimulant use disorder	1
Psychiatric diagnosis	3
HCV	3
Untreated HCV	1
HIV	3
Hepatitis B	2
Chronic liver disease	1
Coronary artery disease	1
Congestive heart failure	1
Self-harm	2
Psychiatric medication at enrolment	1
HAART	1
Injection related complication	1
Not reported	2

*Abbreviations:* HIV, Human immunodeficiency virus; HCV, Hepatitis C virus; HAART, highly active antiretroviral therapy

^a^Multiple categories reported per study

The PROGRESS-PLUS criteria were reported in only two studies (Additional file 1: Appendix 9) [28, 30]. Two studies reported on participants’ gender. Two studies reported on the race of the included patients with White being the majority in both (81.2%, 85%) [28, 30], followed by being Indigenous (12%) and Black (4%) in another [30]. One study included only people experiencing homelessness [30]. Another study reported that 7.3% of the patients were unemployed, 39.7% had a lack of education, and 7.8% were living in poverty [28]. No other PROGRESS-PLUS criteria were reported.

### Risk of bias assessment

**Fig. 2:**
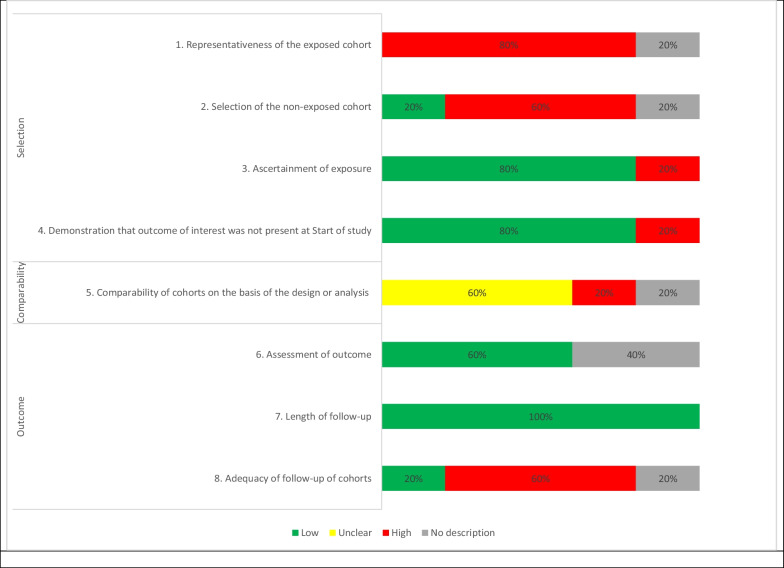
Aggregate Newcastle–Ottawa scale appraisal results for cohort studies (*n* = 5). *Abbreviations:* High, High risk of bias; Low, Low risk of bias; Unclear, Unclear risk of bias

The cohort studies were appraised using the Newcastle–Ottawa scale and judged as having a high risk of bias for not including representative cases in three studies [26, 28, 30], not selecting a representative control group in two studies [26, 30], not ascertaining exposure adequately [29, 31], not adjusting for confounders [28, 29], ensuring misclassification of the outcome did not occur [30, 31], and concerns with loss to follow-up [28–31] (Additional file 1: Appendix 10). The case–control study [27] was appraised using the Newcastle–Ottawa scale and judged as having a high risk of bias for not including representative cases and not selecting a representative control group (Additional file 1: Appendix 11) (Fig. 2).

### Outcome results: research question 1

What are the rates of IE, HCV infection and/or HIV infection in adults exposed to controlled-release hydromorphone compared with the rates of the same infections in people using immediate-release (oral) hydromorphone, injectable hydromorphone, and other controlled-release products globally?

A prospective cohort study was conducted in Calgary, Alberta, including 257 PWID between 2014 and 2017 [29]. Participants were tested for HCV and HIV via serological testing. At baseline, three PWID were HIV-positive and 72 were positive for HCV antibodies. The PWID with positive HCV antibodies (*n* = 6, 9%) were more likely to inject hydromorphone versus PWID with negative HCV antibodies (*n* = 3, 2%, *p* = 0.009). Furthermore, PWID with HCV antibodies were more likely to use non-injection routes of administration for hydromorphone (*n* = 3, 5%) compared with PWID without HCV antibodies (*n* = 0, *p* = 0.002). Three PWID seroconverted during the study and two of these reported opioid use (specific use of hydromorphone was not reported). The authors concluded that more PWID with positive HCV antibodies used hydromorphone through injection and non-injection routes than PWID without HCV antibodies and that they were uncertain whether the HCV infection that occurred in the three PWID who seroconverted during the study was due to hydromorphone use. The study did not differentiate between immediate and controlled-release hydromorphone.

A retrospective cohort study was conducted in Ontario including 60,529 hospital admissions of adult PWID between 2006 and 2015 [26]. Overall, 733 patients diagnosed with IE were matched with 32,576 controls without IE. Of these, 109 (2.8%) patients filled prescriptions with hydromorphone compared with 41 (1.1%) who filled prescriptions for non-hydromorphone opioids (adjusted odds ratio [OR] 2.5, 95% confidence interval [CI] 1.8–3.7, *p* < 0.0001). Furthermore, 21 (1.1%) of the prescriptions were for immediate-release hydromorphone compared with matched PWID who filled prescriptions with non-hydromorphone opioids (adjusted OR 1.7, 95% CI 0.9–3.6, *p* = 0.072). For controlled-release hydromorphone, there were 73 (3.9%) hospital admissions compared with 20 (1.1%) admissions amongst matched PWIDs who filled prescriptions for non-hydromorphone opioids (adjusted OR 3.3, 95% CI 2.1–5.6, *p* < 0.0001). The authors concluded that filling a prescription for controlled-release hydromorphone was associated with a risk of IE that was three times higher than for other opioids.

A retrospective cohort was conducted in Ottawa, Ontario, between 2017 and 2018 including 26 PWID [30]. At baseline, 24 PWID had untreated HCV and eight were living with HIV. No new diagnoses of HCV were observed during the study, whereas one PWID was newly diagnosed with HIV. The injectable hydromorphone dosage increased during the study with 24 PWID who were started on oral controlled-release hydromorphone and two withdrawing from this intervention. One PWID prescribed oral hydromorphone crushed the tablet and injected it instead of taking the medication orally or using the injectable formulation provided. No conclusions were made by the authors between the association of controlled-release hydromorphone and transmission of HCV or HIV amongst PWID.

A case–control study was conducted including 33 cases (adult PWID diagnosed with IE) and 102 controls (adult PWID without IE) admitted to addiction clinics in London, Ontario, between 2016 and 2018 [27]. One-on-one interviews were completed to understand risk factors associated with IE amongst PWID. The most injected drug was controlled-release hydromorphone (91% cases versus 81% controls, *p* = 0.20), which was not statistically significantly different between cases and controls. Heating controlled-release hydromorphone prior to injection was not statistically significantly related to IE. Cases and controls similarly used IDPE (e.g. “cookers”) to prepare the drugs at rates of 50–60%. However, controls were more likely to use IDPE (e.g. Stericup) provided by the provincial government (32%) versus cases (13%, *p* < 0.001), as well as have a greater access to a heating source such as a lighter (58.8%) versus controls (36.4%, *p* = 0.025). Cases were more likely to use IDPE cookers that were not provided as examples on the survey (48.5%), such as spoon, glass bottle, or shot glass compared with controls (11.8%, *p* < 0.001). The authors concluded that a significant risk in IE for PWID using controlled-release hydromorphone was not observed and that use of IDPE provided by the government (e.g. Stericup) and lighters to prepare the drugs might be protective against IE.

#### Association study

A retrospective cohort study was conducted using health unit data on HCV infection and opioid prescription data from Ontario, Canada, in 2016 [28]. There were 4,079 new diagnoses of HCV infection and an average of 1.8 kg per 10,000 population of hydromorphone controlled-release prescription opioid sales. The study found that an increase in hydromorphone controlled-release dispensing rate was a stronger predictor of HCV incidence compared with all opioids overall (standardized risk ratio 1.17, *p* < 0.001). The authors concluded that prescription of controlled-release hydromorphone is contributing to HCV transmission in Ontario.

### Outcome results: research question 2

What are the characteristics of adults exposed to controlled-release hydromorphone who experienced IE, HCV infection and/or HIV infection, including previous treatment or hospitalization for opioid-related harms? None of the included studies reported results specific to previous treatment or hospitalization for opioid-related harms.

## Discussion

We conducted a comprehensive systematic review on the potential risk of HCV, IE, and HIV infection amongst adults exposed to controlled-release hydromorphone. Only five relevant studies were identified, which indicates a lack of evidence in this area. Although some studies demonstrated a potential association between the use of controlled-release hydromorphone and HCV, IE, and HIV incidence, all but two studies were based on retrospective data [29]. One retrospective study did not find an association with IE [27]. As well, two studies focused on hydromorphone overall and not the controlled-release formulation, which makes interpretation difficult. None of the studies specifically reported on associations with HIV.

Injection risks with controlled-release hydromorphone may be related to sharing of IDPE, which suggests interventions that could decrease this harm. The process for preparing HCR for injection involves removing the substance from the capsule, crushing it in a metal cooker, and adding sterile water. One study suggested that heating controlled-release hydromorphone prior to injection may decrease the risk of IE. Another found that the use of a particular cooker (the Stericup) may be protective against IE.

Our systematic review identified several gaps in the literature. All studies were conducted in Canada, indicating a gap in the literature from other countries, especially low- and middle-income countries, as well as in countries with high opioid usage, such as the USA. Furthermore, only two studies reported on the PROGRESS-PLUS criteria [23], which can be used to examine equity issues in research. Future research in this area should report on all the PROGRESS-PLUS criteria so that targeted interventions can be developed to address social determinants of health at the same time as addressing harm reduction.

There are limitations to our systematic review that are worth noting. One is the lack of available evidence on this topic, which limits our interpretation of results. We also excluded adolescents from our review and use of controlled-release hydromorphone might occur in this population. Due to the limited evidence in this area, we were more liberal in our inclusion of studies compared with our registered protocol and included studies examining hydromorphone overall and not focused on the controlled-release formulation. We were also unable to conduct GRADE assessments for certainty of evidence. These are deviations from our protocol. Another limitation is the risk of bias of the included studies; particularly regarding the lack of controlling for confounding across the studies. The strengths of our systematic review included following the methodologically rigorous Cochrane handbook and the PRISMA 2020 reporting guidance.

## Conclusions

Very few studies have examined the risk of IE, HCV, and HIV infection after exposure to controlled-release hydromorphone. Very low-quality and scant evidence suggests uncertainty around the risks of blood-borne infections, such as HCV and IE to PWID using this medication.

## Supplementary Information

**Additional file 1.** Supplementary file and appendices

## Data Availability

The dataset(s) supporting the conclusions of this article is(are) included within the article (and its additional file(s)).

## Abbreviations

CADTH: Canadian Agency for Drugs and Technologies in Health
EPOC: Effective Practice and Organization of Care
HCV: Hepatitis C virus
HIV: Human immunodeficiency virus
IDPE: Injection drug preparation equipment
IE: Infective endocarditis
PRESS: Peer Review of Electronic Search Strategies
PRISMA-P: Preferred reporting items for systematic reviews and meta-analysis for Protocols
PWID: People who inject drugs
WHO: World Health Organization
