# Supplementary material for: Controlled-release hydromorphone and risk of infection in adults: a systematic review
Source: Harm Reduct J. 2023 Apr 28;20:60. doi: 10.1186/s12954-023-00788-9 (PMC10142404; doi:10.1186/s12954-023-00788-9)
Supplement: Supplementary file 1 — Additional file 1. Supplementary file and appendices [file 12954_2023_788_MOESM1_ESM.docx]

Hydromorphone Controlled Release: Systematic Review – Appendices

Contents

[Appendix 1 – Database Search Strategies 2](#_Toc109823005)

[Appendix 2 - Grey Literature Sources 3](#_Toc109823006)

[Appendix 3 – L1 Screening Form for Titles and Abstracts 4](#_Toc109823007)

[Appendix 4 – L2 Screening Form for Full-Text Articles 6](#_Toc109823008)

[Appendix 5 – Data Abstraction Form 9](#_Toc109823009)

[Appendix 6 – Excluded Studies 18](#_Toc109823010)

[Appendix 7 – Study Characteristics 19](#_Toc109823011)

[Appendix 8 – Patient Characteristics 1 20](#_Toc109823012)

[Appendix 9 – Patient Characteristics 2 21](#_Toc109823013)

[Appendix 10 – Newcastle Ottawa Scale for Cohort Studies 22](#_Toc109823014)

[Appendix 11 – Newcastle Ottawa Scale for Case-Control and Case-Test Negative Studies 23](#_Toc109823015)

# Appendix 1 – Database Search Strategies

**Database: Ovid MEDLINE(R) ALL <1946 to September 14, 2021>**
1     Hydromorphone/
2     (Hydromorph* or hymorphan or HMC or dihydromorphinone or dihydromorphone or dimorphone or dolonovag or laudacon or palladon* or jurnista or dilaudid or dilaudid-HP or diladid or dilaudid−5 or exalgo or Hydromorphone-CR or Hydromorphone-IR or hydrostat or Infumorph or Kadian or Kapanol or Oros or DiMo or novolaudon or biomorphyl or cofalaudid or hydal or laudaconum or novolaudon or opidol or rexaphon or semcox or sophidone).tw,kf.

3     or/1-2
4     ((sustained or controlled or extended or slow or prolong* or time or slow or low) adj2 release*).tw,kf.
5     (Sustained-Release or controlled-release or extended-release or slow-release or CR).tw,kf.
6     Delayed-Action Preparations/ or Administration, Intravenous/
7     ad.fs.
8     SROM.tw,kf.
9     Substance Abuse, Intravenous/ or Opioid-Related Disorders/
10     or/4-9
11     3 and 10
12     exp animals/ not humans.sh.
13     11 not 12

**Database: Embase Classic+Embase <1947 to 2021 September 14>**
1     exp hydromorphone/
2     (Hydromorph* or hymorphan or HMC or dihydromorphinone or dihydromorphone or dimorphone or dolonovag or laudacon or palladon* or jurnista or dilaudid or dilaudid-HP or diladid or dilaudid-5 or exalgo or Hydromorphone-CR or Hydromorphone-IR or hydrostat or Infumorph or Kadian or Kapanol or Oros or DiMo or novolaudon or biomorphyl or cofalaudid or hydal or laudaconum or novolaudon or opidol or rexaphon or semcox or sophidone).tw.
3     1 or 2
4     ((sustained or controlled or extended or slow or prolong* or time or slow or low) adj2 release*).tw.
5     (Sustained-Release or controlled-release or extended-release or slow-release or CR).tw.
6     SROM.tw.
7     exp controlled release formulation/
8     intravenous drug administration/
9     substance abuse/
10     opiate addiction/
11     or/4-10
12     3 and 11 *

**Database: EBM Reviews - Cochrane Database of Systematic Reviews <2005 to September 9, 2021>, EBM Reviews - ACP Journal Club <1991 to August 2021>, EBM Reviews - Database of Abstracts of Reviews of Effects <1st Quarter 2016>, EBM Reviews - Cochrane Clinical Answers <August 2021>, EBM Reviews - Cochrane Central Register of Controlled Trials <August 2021>, EBM Reviews - Cochrane Methodology Register <3rd Quarter 2012>, EBM Reviews - Health Technology Assessment <4th Quarter 2016>, EBM Reviews - NHS Economic Evaluation Database <1st Quarter 2016>**--------------------------------------------------------------------------------
1     (Hydromorph* or hymorphan or HMC or dihydromorphinone or dihydromorphone or dimorphone or dolonovag or laudacon or palladon* or jurnista or dilaudid or dilaudid-HP or diladid or dilaudid-5 or exalgo or Hydromorphone-CR or Hydromorphone-IR or hydrostat or Infumorph or Kadian or Kapanol or Oros or DiMo or novolaudon or biomorphyl or cofalaudid or hydal or laudaconum or novolaudon or opidol or rexaphon or semcox or sophidone).tw.
2     ((sustained or controlled or extended or slow or prolong* or time or slow or low) adj2 release*).tw.
3     (Sustained-Release or controlled-release or extended-release or slow-release or CR or SROM).tw.
4     1 and (2 or 3)

# Appendix 2 - Grey Literature Sources

AHRQ: <https://www.ahrq.gov/>

ASAM: <https://www.asam.org/>

BCCSU: <https://www.bccsu.ca/>

CADTH: <https://www.cadth.ca/about-cadth>

CenterWatch: <https://www.centerwatch.com/clinical-trials/listings/>

CIHI: <https://www.cihi.ca/en>

ClinicalTrials.gov: <https://www.clinicaltrials.gov/>

CMA Infobase: <https://joulecma.ca/cpg/homepage>

CRD York: <https://www.crd.york.ac.uk/CRDWeb/>

Euroscan: <https://www.euroscan.org/index.php/en/>

Health Canada: <https://www.canada.ca/en/health-canada.html>

HQO:-<https://www.hqontario.ca/evidence/publications-and-ohtac-recommendations>

International Clinical Trials Registry Platform (ICTRP): <https://trialsearch.who.int/>

INAHTA: <http://www.inahta.org/publications/>

INESS: <https://www.inesss.qc.ca/en/publications/publications.html>

ISPOR: <https://www.ispor.org/>

NTIS: <https://www.ntis.gov>

OpenGrey: <http://www.opengrey.eu>

PHAC: <https://www.canada.ca/en/public-health.html>

Statistics Canada: <https://www.statcan.gc.ca/en/start>

Trip database: <http://www.tripdatabase.com>

WHO: <http://www.euro.who.int/en/data-and-evidence/evidence-informed-policy-making/publications/by-keyword>

# Appendix 3 – L1 Screening Form for Titles and Abstracts

**Hydromorphone Controlled Release Systematic Review – Level 1 Cheat Sheet**

*Version 9*

**Review objective:** To identify risk of IE, HCV infection and/or HIV infection in individuals exposed to HCR compared with other opioids and determine the characteristics of HCR users.

**Review questions:**

1. What are the rates of IE, HCV infection and/or HIV infection in adults exposed to HCR (either through prescription, unregulated use or injection – i.e., people who inject drugs or PWID) compared to the rates of the same infections in users of immediate release (oral) hydromorphone, injectable hydromorphone, and users of other controlled-release products globally?
2. What are the characteristics of adult HCR users who experienced IE, HCV infection and/or HIV infection, including previous treatment or hospitalization for opioid-related harms?

**Synthesi.SR link:** <https://breakthroughkt.ca/> **Project name: *Hydromorphone SR*** ***– L1***

NOTES

- If you select ‘NO’ to any screening question, the study will be excluded, and the remaining questions can be skipped.
- If you select ‘UNCLEAR’ or ‘YES’ to all questions, the study will be included for Level 2 screening.
- If a study only has a title, select ‘UNCLEAR’ for all questions if it mentions hydromorphone OR opioids.
- Hydromorphone extended-release tablets are indicated for the management of pain in patients who are opioid-tolerant severe enough to require daily, around the clock, long term opioid treatment, when first line treatments have failed/been exhausted.

| **Question 1:** | **Does the study involve persons who have used Hydromorphone Controlled Release (HCR) through prescription, unregulated use, or injection^1^?** | | |
| --- | --- | --- | --- |
| **RESPONSE** | **YES** | **NO** | **UNCLEAR** |
| Guidance notes | Used HCR^2,3^  ****must be controlled release, extended release, slow release, etc. versions**** | No HCR use reported  Immediate/regular release versions  Liquid/solution versions  Powders  Syrups  Intrathecal, intramuscular, epidural, nerve blocks, nasal mucoadhesive, injection | If HCR use is unclear, vague, or does not specify release type  Analgesics  Opioids |
| ^1^ Notes: injection may be referred to as “people who inject drugs” or PWID, injection drug user, intravenous drug user or IDU.  ^2^ Examples of drug/brand names include: hydromorphone extended-release capsules/tablets, Dilaudid, Dilaudid-5, Exalgo, Palladone, Hydromorphone, Hydromorphone hydrochloride, Dihydromorphinone, Laudacon, Jurnista, Novolaudon.  ^3^Also applies if the patients are taking HCR and fentanyl patch simultaneously | | | |

| **Question 2:** | **Does the study involve adults aged 18 years and older?** | | |
| --- | --- | --- | --- |
| **RESPONSE** | **YES** | **NO** | **UNCLEAR** |
| Guidance notes | Age ≥ 18 years | Age below 18 years | Age not clearly reported |

| **Question 3:** | **Is the study a relevant study design?** | | | |
| --- | --- | --- | --- | --- |
| **RESPONSE** | **YES** | **NO** | **NO but flag as relevant** | **UNCLEAR** |
| Guidance notes | Randomized controlled trial  Non-RCT  Quasi-experimental  Interrupted time series  Controlled before-after  Uncontrolled before-after  Case control  Cohort | Cross sectional  Case series  Case reports  Qualitative  Opinion pieces (editorials, commentary, letters)  Pharmacokinetic studies | Systematic review  Conference abstract  Trial Protocol  Non-English article | Study design not clearly reported |
|  | **Note:** *studies MUST have a control or comparator to be eligible for inclusion* |  | **Note:** *please only flag if YES or UNCLEAR to all other questions* |  |

# Appendix 4 – L2 Screening Form for Full-Text Articles

**Hydromorphone Controlled Release Systematic Review – Level 2 Cheat Sheet**

*Version 3*

**Review objective:** To identify risk of infective endocarditis (IE), hepatitis C virus (HCV) infection and/or human immunodeficiency virus (HIV) infection in individuals exposed to HCR compared with other opioids and determine the characteristics of HCR users.

**Review questions:**

1. What are the rates of IE, HCV infection and/or HIV infection in adults exposed to HCR (either through prescription, unregulated use or injection – i.e., people who inject drugs or PWID) compared to the rates of the same infections in users of immediate release (oral) hydromorphone, injectable hydromorphone, and users of other controlled-release products globally?
2. What are the characteristics of adult HCR users who experienced IE, HCV infection and/or HIV infection, including previous treatment or hospitalization for opioid-related harms?

**Synthesi.SR link:** <https://breakthroughkt.ca/> **Project name: *Hydromorphone SR*** ***– L2***

NOTES

- If you select ‘NO’ to any screening question, the study will be excluded, and the remaining questions can be skipped.
- If you select ‘UNCLEAR’ or ‘YES’ to all questions, the study will be included for data abstraction.
- Hydromorphone extended-release tablets are indicated for the management of pain in patients who are opioid-tolerant severe enough to require daily, around the clock, long term opioid treatment, when first line treatments have failed/been exhausted.

| **Question 1:** | **Does the study involve persons who have used Hydromorphone Controlled Release (HCR) through prescription, unregulated use, or injection^1,2^?** | | |
| --- | --- | --- | --- |
| **RESPONSE** | **YES** | **NO** | **UNCLEAR** |
| Guidance notes | Used HCR^3,4^  ****must be controlled release, extended release, slow release, OROS, etc. versions**** | No HCR use reported  Immediate/regular release versions  Liquid/solution versions  Powders  Syrups  Intrathecal, intramuscular, epidural, nerve blocks, nasal mucoadhesive, injection, subcutaneous, intravenous, patient-controlled analgesia (PCA) pump^5^ | If HCR use is unclear, vague, or does not specify release type  Analgesics  Opioids |
| ^1^ Notes: injection may be referred to as “people who inject drugs” or PWID, injection drug user, intravenous drug user or IDU.  ^2^ If it is clear that the formulation is extended release regardless of delivery method, include. If the formulation is unclear and the delivery method is in the NO column, exclude.  ^3^ Examples of drug/brand names include: hydromorphone extended-release capsules/tablets, Dilaudid, Dilaudid-5, Exalgo, Palladone, Hydromorphone, Hydromorphone hydrochloride, Dihydromorphinone, Laudacon, Jurnista, Novolaudon.  ^4^ Also applies if the patients are taking HCR and fentanyl patch simultaneously  ^5^UNLESS it is made clear that the version is controlled release, extended release, slow release, etc. | | | |

| **Question 2:** | **Does the study involve adults aged 18 years and older?** | | |
| --- | --- | --- | --- |
| **RESPONSE** | **YES** | **NO** | **UNCLEAR** |
| Guidance notes | Age ≥ 18 years | Age below 18 years | Age not clearly reported |

| **Question 3:** | **Does the study report on the outcomes^6^ of interest?** | | |
| --- | --- | --- | --- |
| **RESPONSE** | **YES** | **NO** | **UNCLEAR** |
| Guidance notes | Infective endocarditis (IE)  Human immunodeficiency virus (HIV)  Hepatitis C virus (HCV) | No outcome identified | Outcome is not clearly reported |
| ^6^ Outcomes of interest are **incident** cases of ANY of IE, HIV, HCV --- incident cases are newly diagnosed cases of a disease vs a prevalent case which would be people who may have had the disease for some time. | | | |

| **Question 4:** | **Does the study involve an opioid comparator?** | | |
| --- | --- | --- | --- |
| **RESPONSE** | **YES** | **NO** | **UNCLEAR** |
| Guidance notes | Immediate release hydromorphone  Injectable hydromorphone  Other controlled-release opioids^7^  Other immediate release opioids^7^ | No comparator identified | Comparator is not clearly reported |
| ^7^Examples of other opioid comparators could include: morphine, fentanyl, oxycodone, etc. | | | |

| **Question 5:** | **Is the study a relevant study design?** | | | |
| --- | --- | --- | --- | --- |
| **RESPONSE** | **YES** | **NO** | **NO but flag as relevant** | **UNCLEAR** |
| Guidance notes | Randomized controlled trial  Non-RCT  Quasi-experimental  Interrupted time series  Controlled before-after  Uncontrolled before-after  Case control  Cohort | Cross sectional  Case series  Case reports  Qualitative  Opinion pieces (editorials, commentary, letters)  Pharmacokinetic studies | Systematic review  Conference abstract  Trial Protocol  Non-English article | Study design not clearly reported |
|  | **Note:** *studies MUST have a control or comparator to be eligible for inclusion* |  | **Note:** *please only flag if YES or UNCLEAR to all other questions* |  |

# Appendix 5 – Data Abstraction Form

**Hydromorphone Controlled Release**

DATA CHARTING CHEAT SHEET

Version 5 – March 7^th^, 2022

**Study characteristics**

| Excel column | Description |
| --- | --- |
| REFID | Enter the unique reference identification number of the study  Example: 857002 |
| Reviewer Initials | Enter your initials  Example: AP |
| Last name of first author | Type in the last name of the first author  Example: Smith |
| Year of publication | Enter the year the study was published |
| Study title | Enter the title of the article |
| Journal name | Enter the name of the journal that the article was published in |
| Country of conduct | List the country where the study was conducted (do not enter in cities)  If the trial is a multi-site trial, please list all the countries separated by commas  If the country of conduct is not clear, use the country from the first author’s affiliation.  Example: USA, Canada, and Australia |
| Publication type | Select appropriate publication type from the drop down menu.  Options include: Journal article, thesis, conference abstract, trial protocol, non-English article |
| Study design | Select appropriate study design from the drop down menu.  Options include: RCT, non-RCT, quasi-experimental, interrupted time series, controlled before-after, uncontrolled before-after, case control, cohort, unclear, NR  *If you select “other” please specify design in the comments column* |
| Overall sample size | Report the total number of participants in the study (across all treatment arms) |
| Study duration | Report the duration of the study in months (duration of intervention+follow-up)  Example: If the intervention is 6 months, and they have 6 months of follow-up, we would report “12” for this data point. |
| Study setting | Enter text describing the setting in which the study was conducted.  Example: An urban community in Canada |
| Multi-center vs single site | Enter whether the study was conducted at a single site or was it spread out over multiple centers |
| Comments | Enter any details that you feel are relevant and are not captured elsewhere |

**Patient characteristics**

| Excel column | Description |
| --- | --- |
| Reported eligibility criteria or description of participants | Enter any listed eligibility criteria or description of the participant sample or population as reported in the study |
| Reported exclusionary criteria for participant population | Enter any listed exclusion criteria as reported in the study. |
| Participant living environment | Enter text describing the participants’ living environment  Example: Experiencing homelessness |
| Treatment Arm #1 | |
| Age value | Enter the overall age of all patients if reported  Example: 71.2 |
| Reported as: | Select type of age value from the dropdown menu  Options include: mean, median, range, unclear, NR |
| Variance value | Enter the value of the variance reported.  Example: SD: 14.3; Range: 67-89 |
| Variance type | Select type of value from the dropdown menu.  Options include: standard deviation (SD), standard error (SE), standard error of the mean (SEM), interquartile range (IQR), 95% CI, range, unclear, NR |
| % female | Enter the overall percentage of females in the study (across treatment arms)  If necessary, calculate using the following:  Calculation: *[(#Females1+#Females2)/(Sample1+Sample2)]*100* |
| % male | Enter the overall percentage of males in the study (across treatment arms)  *If necessary, calculate as specified above* |
| % other | Enter the overall percentage of individuals who do not identify as male or female in the study (across treatment arms)  *If necessary, calculate as specified above* |
| % of participants who are injection / intravenous drug users | Enter the overall percentage of individuals who identify as injection / intravenous drug users |
| % of participants obtaining the drug through prescription | Enter the overall percentage of individuals who obtained the drug through prescription |
| % of participants obtaining the drug through unregulated sources | Enter the overall percentage of individuals who obtained the drug through unregulated sources |
| List of comorbidities | List any relevant comorbidities with the percentage of participants who have them  Example: Depression (23%), Anxiety (15.2%), etc. |
| PROGRESS items | Enter any relevant details from the PROGRESS framework not covered elsewhere (e.g. race/ethnicity/culture/language, occupation, religion, education, socioeconomic status, social capital). |
| Comments | Enter any relevant details that are not captured elsewhere |
| Treatment Arm #2 | |
| Age value | Enter the overall age of all patients if reported  Example: 71.2 |
| Reported as: | Select type of age value from the dropdown menu  Options include: mean, median, range, unclear, NR |
| Variance value | Enter the value of the variance reported.  Example: SD: 14.3; Range: 67-89 |
| Variance type | Select type of value from the dropdown menu.  Options include: standard deviation (SD), standard error (SE), standard error of the mean (SEM), interquartile range (IQR), 95% CI, range, unclear, NR |
| % female | Enter the overall percentage of females in the study (across treatment arms)  If necessary, calculate using the following:  Calculation: *[(#Females1+#Females2)/(Sample1+Sample2)]*100* |
| % male | Enter the overall percentage of males in the study (across treatment arms)  *If necessary, calculate as specified above* |
| % other | Enter the overall percentage of individuals who do not identify as male or female in the study (across treatment arms)  *If necessary, calculate as specified above* |
| % of participants who are injection / intravenous drug users | Enter the overall percentage of individuals who identify as injection / intravenous drug users |
| % of participants obtaining the drug through prescription | Enter the overall percentage of individuals who obtained the drug through prescription |
| % of participants obtaining the drug through unregulated sources | Enter the overall percentage of individuals who obtained the drug through unregulated sources |
| List of comorbidities | List any relevant comorbidities with the percentage of participants who have them  Example: Depression (23%), Anxiety (15.2%), etc. |
| PROGRESS items | Enter any relevant details from the PROGRESS framework not covered elsewhere (e.g. place of residence, race/ethnicity/culture/language, occupation, gender/sex, religion, education, socioeconomic status, social capital). |
| Comments | Enter any relevant details that are not captured elsewhere |
| *Note: If a study has more than two treatment arms, please copy the Treatment Arm 2 columns and insert them immediately after the existing Treatment Arm 2 columns.* | |

**Treatment arms**

NOTE: This tab is only applicable to studies with multiple treatment arms (intervention studies, cohort studies, case control). If there are more than two treatment arms see directions below.

| Excel column | Description |
| --- | --- |
| Intervention duration | Indicate duration of the intervention provided in months  Enter NA if this is not an intervention study |
| Follow-up duration | Indicate the longest duration of follow-up for which results are reported (in months) |
| Treatment arm 1 arm name | Enter the name of the intervention/treatment arm/group provided  Example: Hydromorphone controlled release |
| Treatment arm 1 description | Enter a description of what the intervention entailed  (copy and paste description from paper) |
| Treatment arm 1 sample size | Specify the number of participants receiving this intervention |
| Dose and formulation | Enter the dose prescribed or taken, as well as the formulation (e.g. 4mg hydromorphone controlled release, oral tablets) |
| Timing of treatment | Enter how often the dose was prescribed or taken |
| Concurrent medication or unregulated drug use | Enter any additional simultaneous drug use, prescribed or illicit (e.g. fentanyl). |
| Treatment arm 2 name | Enter the name of the comparator group  Example: Any other opioid |
| Treatment arm 2 description | Enter a description of what the intervention/treatment arm entailed  (copy and paste description from paper) |
| Treatment arm 2 sample size | Specify the number of participants in the comparator group |
| Dose and formulation | Enter the dose prescribed or taken, as well as the formulation (e.g. 4mg hydromorphone immediate release, oral tablets) |
| Timing of treatment | Enter how often the dose was prescribed or taken |
| Concurrent medication or unregulated drug use | Enter any additional simultaneous drug use, prescribed or unregulated (e.g. fentanyl). |
| *Note: If a study has more than two treatment arms, please copy the Treatment Arm 2 columns (Columns K – P in this tab) and insert them immediately after the existing Treatment Arm 2 columns.* | |

Notes:

*If the study does not report on any relevant outcomes that can be captured in this tab (i.e. with baseline and follow-up values), please enter the refID and grey highlight the entire row*

If a study has multiple outcomes, please report them on separate rows in this tab

Categorical outcomes can also be captured in this tab by specifying “number” under “Outcome value type”, and indicating variance values as NA (not applicable)

**Quantitative Outcomes**

| Excel column | Description |
| --- | --- |
| RefID | Enter the refID of the study being abstracted |
| Initials | Enter your initials |
| Outcome measure | Enter the name of the outcome being measured  Example: Incidence of HIV/HCV/IE |
| Outcome measure definition | Enter the definition of how the outcome is measured. |
| Outcome measurement time point | Select the time point for which you are abstracting results from the dropdown menu  Options include: Baseline, Follow-up  *Note: For each outcome measure, the baseline values and follow-up values will be captured in separate rows*  *Please capture follow-up duration in the Comments column* |
| Duration of follow up | Time to the longest duration of follow up in months |
| Treatment arm 1 name | Copy the Treatment arm 1 name that you entered in the previous tab (if applicable |
| Sample size | Enter the sample size for the intervention arm  (at the time point specified – i.e. baseline or follow-up) |
| Outcome value | Enter the outcome value |
| Outcome value type | Select the value type from the dropdown menu  Options include: number, mean, median, range, unclear, NR |
| Variance value | Enter the variance value |
| Variance type | Select the variance type from the dropdown menu  Options include: NA (not applicable) standard deviation (SD), standard error (SE), standard error of the mean (SEM), interquartile range (IQR), 95% CI, 90% CI, range, unclear, NR |
| Was the exposure as a result of prescription access or unregulated access to the drug? | Select from the dropdown menu  Options include: prescription access, unregulated access, NR (not reported) |
| Treatment arm 2 name | Copy the Treatment arm 2 name that you entered in the previous tab |
| Sample size | Enter the sample size for the comparator arm  (at the time point specified – i.e. baseline or follow-up) |
| Outcome value | Enter the outcome value |
| Outcome value type | Select the value type from the dropdown menu  Options include: number, mean, median, range, unclear, NR |
| Variance value | Enter the variance value |
| Variance type | Select the variance type from the dropdown menu  Options include: standard deviation (SD), standard error (SE), standard error of the mean (SEM), interquartile range (IQR), 95% CI, 90% CI, p-value, range, unclear, NR |
| Was the exposure as a result of prescription access or unregulated access to the drug? | Select from the dropdown menu  Options include: prescription access, unregulated access, NR (not reported) |
| *Note: If a study has more than two treatment arms, please copy Columns K-P in Study Outcomes tab and insert them immediately after the existing columns* | |
| Text description or interpretation of results | Copy and paste any description interpreting the results from the text of the paper |
| Comments | Enter any relevant comments that are not captured elsewhere |

**Statistical Model data**

Notes: This tab is to capture any data related to correlation or regression models that link our variables of interest

| Excel column | Description |
| --- | --- |
| RefID | Enter the refID of the study being abstracted |
| Initials | Enter your initials |
| Variable A | Enter the independent variable related to HCR use  Example: Filled a prescription in the last 12 months |
| Outcome measure for variable A | Enter how variable A was measured  Example: Reported filling a prescription in the last 12 months |
| Variable B | Enter the dependent variable related to incident cases of HIV/HCV/IE  Example: Diagnosed with HIV/HCV/IE since the beginning of the study |
| Outcome measure for variable B | Enter how variable B was measured  Example: Blood test |
| Correlation or regression coefficient | Enter the correlation or regression coefficient value |
| Correlation or regression coefficient type | Enter the type of coefficient  Example: OR (odds ratio) |
| Variance value | Enter the variance value |
| Variance type | Select the variance type from the dropdown menu  Options include: standard deviation (SD), standard error (SE), standard error of the mean (SEM), 95% CI, range, interquartile range (IQR), unclear, NR, NA |
| p-value | Enter the p-value – may be denoted by “***” at the bottom of the table |
| Statistically significant? | Select from the dropdown menu  Options include: yes, no, NR |
| Analysis method | Enter in the analysis methods used  Example: Multiple linear regression |
| Confounders / variables controlled for in model | List any confounders or variables which were controlled for in the model. Copy and paste any description of confounders/controls from the paper. |
| Text description or interpretation of relationship between Variable A and B | Copy and paste any description interpreting the results from the text of the paper |
| Comments | Enter any relevant comments that are not captured elsewhere |

# Appendix 6 – Excluded Studies

| **Study ID number** | **Reference** | **Reason for exclusion** |
| --- | --- | --- |
| 1075045 | Fischer, B., Cruz, M. F., & Rehm, J. (2006). Illicit opioid use and its key characteristics: a select overview and evidence from a Canadian multisite cohort of illicit opioid users (OPICAN). The Canadian Journal of Psychiatry, 51(10), 624-634. | This article is a review of the evidence from the OPICAN study, not a primary article. |
| NR | Ho, J., DeBeck, K., Milloy, M. J., Dong, H., Wood, E., Kerr, T., & Hayashi, K. (2018). Increasing availability of illicit and prescription opioids among people who inject drugs in a Canadian setting, 2010–2014. *The American journal of drug and alcohol abuse*, *44*(3), 368-377. | This article focuses on the availability of Dilaudid. |
| 1075707 | Ross, D., Lo, F., McKim, R., & Allan, G. M. (2008). A primary care/multidisciplinary harm reduction clinic including opiate bridging. Substance use & misuse, 43(11), 1628-1639. | Dialuid is used by 1 participant, the outcome was reported as "injection" vs "non-injection" drug users. |
| 1077286 | Weir, M. A., Slater, J., Jandoc, R., Koivu, S., Garg, A. X., & Silverman, M. (2019). The risk of infective endocarditis among people who inject drugs: a retrospective, population-based time series analysis. Cmaj, 191(4), E93-E99. | This article focuses on trends in infective endocarditis related to injection drug use. |
| Greylit_251 | Weir, M. A., Slater, J., Jandoc, R., Koivu, S., Garg, A. X., & Silverman, M. (2019). The risk of infective endocarditis among people who inject drugs: a retrospective, population-based time series analysis. Cmaj, 191(4), E93-E99. | Duplicate of study above – 1077286 |
| Greylit_99 | Silverman, M., Slater, J., Jandoc, R., Koivu, S., Garg, A. X., & Weir, M. A. (2020). Hydromorphone and the risk of infective endocarditis among people who inject drugs: a population-based, retrospective cohort study. The Lancet Infectious Diseases, 20(4), 487-497. | Duplicate of included study – 1076662 |

# Appendix 7 – Study Characteristics

| **Author, year** | **Study title, year(s) of study conduct** | **Journal name** | **Country** | **Study design** | **Study duration (months)** | **Setting** | **Multi-centre vs. single site** |
| --- | --- | --- | --- | --- | --- | --- | --- |
| Zietara, 2020 | Sociodemographic risk factors for hepatitis C virus infection in a prospective cohort study of 257 persons in Canada who inject drugs, 2014-2017 | International Journal of Drug Policy | Canada | Cohort | 64 | Urban inner-city clinics and homeless shelters in Calgary (ie, Calgary Drop In Centre, Calgary Alpha House and Calgary Urban Project Society) | Multi-centre |
| Silverman, 2020 | Hydromorphone and the risk of infective endocarditis among people who inject drugs: a population-based, retrospective cohort study, 2016-2015 | The Lancet Infectious Diseases | Canada | Cohort | 113 | Ontario, Canada | Multi-centre |
| Harris, 2021 | Outcomes of Ottawa, Canada’s Managed Opioid Program (MOP) where supervised injectable hydromorphone was paired with assisted housing, 2017-2018 | International Journal of Drug Policy | Canada | Cohort | 12 | Ottawa Inner City Health - Managed Opioid Program (MOP) | Single site |
| Shah, 2020 | Risk factors of infective endocarditis in persons who inject drugs, 2016-2018 | Harm Reduction Journal | Canada | Case control | 23 | Three tertiary care centers, outpatient clinics and addiction clinics in London, Ontario Canada | Multi-centre |
| Meyer, 2020 | New hepatitis C diagnoses in Ontario, Canada are associated with the local prescription patterns of a controlled-release opioid, 2016 | Journal of Viral Hepatitis | Canada | Cohort | 12 | Health units in Ontario, Canada | Multi-center |

# Appendix 8 – Patient Characteristics 1

| **DEMOGRAPHIC DATA** | | | | | | | |
| --- | --- | --- | --- | --- | --- | --- | --- |
| **Author, year** | **Overall sample size** | **Overall age (years)** | **Overall age (type)** | **Overall age variance (value)** | **Overall age variance (type)** | **% female** | **% male** |
| Zietara, 2020 | 257 | NR | NR | NR | NR | NR | NR |
| Silverman, 2020 | 3790 | 44.6 | mean | 8.4 | SD | 42.5 | 57.5 |
| Harris, 2021 | 26 | 36 | median | 29 - 47 | IQR | 54 | 46 |
| Shah, 2020 | 135 | NR | NR | NR | NR | NR | NR |
| Meyer, 2020 | NR | NR | NR | NR | NR | NR | NR |
| NR: Not reported  SD: Standard deviation  IQR: Inter-quartile range | | | | | | | |

# Appendix 9 – Patient Characteristics 2

| **DEMOGRAPHIC DATA CONT’D** | | | | | |
| --- | --- | --- | --- | --- | --- |
| **Author, year** | **Participants who are PWID (%)** | **Participants obtaining the drug through unregulated sources (%)** | **Participants obtaining the drug through prescription (%)** | **List of comorbidities [comorbidity 1 (%), etc.]** | **PROGRESS items^1^** |
| Zietara, 2020 | NR | NR | NR | NR | NR |
| Silverman, 2020 | 100 | 100 | 0 | Alcoholism (5.5%), Bipolar disorder (1.3%), Chronic liver disease (11.3%), Congestive heart failure (5.0%), Coronary artery disease (0.9%), Depression or anxiety disorder, or both (22.9%), Hepatitis B (<6%), Hepatitis C (24.9%), HIV (7.6%), Schizophrenia or other psychotic disorder (0.9%), Self harm (13.4%) | NR |
| Harris, 2021 | 100 | 0 | 100 | Alcohol use disorder (31%), Stimulant use disorder (96%), Psychiatric diagnosis (100%), Psychiatric medication at enrollment (54%), Injection-related complication, (73%), Untreated HCV, (92%), HIV infection (31%), HAART (highly active antiretroviral therapy) (50%) | Ethnicity: White (85%), Indigenous (12%), Black (4%); Homelessness 100% |
| Shah, 2020 | 100 | NR | NR | NR | NR |
| Meyer, 2020 | NR | 100 | 0 | NR | White race/ethnicity (mean: 81.2%), Unemployment (mean: 7.3%), Lack of Education (mean: 39.7%), Poverty (mean:7.8%) |
| NR: Not reported | | | | | |

^1^O'Neill J, Tabish H, Welch V, Petticrew M, Pottie K, Clarke M, Evans T, Pardo Pardo J, Waters E, White H, Tugwell P. [Applying an equity lens to interventions: using PROGRESS ensures consideration of socially stratifying factors to illuminate inequities in health](http://www.jclinepi.com/article/S0895-4356(13)00334-X/abstract). Journal of Clinical Epidemiology. 2014, 67 (1), pg. 56-64. doi:10.1016/j.jclinepi.2013.08.005

# Appendix 10 – Newcastle Ottawa Scale for Cohort Studies

| **Author, year** | **Representativeness of the exposed cohort** | **Selection of the non-exposed cohort** | **Ascertainment of exposure** | **Demonstration that outcome of interest was not present at Start of study** | **Comparability of cohorts on the basis of the design or analysis** | **Outcome** | | **Adequacy of follow-up of cohorts** |
| --- | --- | --- | --- | --- | --- | --- | --- | --- |
|  |  |  |  |  |  | **Assessment of outcome** | **Length of follow-up** |  |
| Harris, 2021 | No star | No star | Star | Star | Star | Not reported | Star | No star |
| Harris, 2021 | No star | No star | Star | Star | Star | Not reported | Star | No star |
| Silverman, 2020 | No star | No star | Star | Star | Star | Star | Star | Star |
| Zietara, 2020 | Not reported | Not reported | No star | Star | No star | Star | Star | No star |
| Meyer, 2020 | No star | Star | Star | No star | Not reported | Star | Star | Not reported |

# Appendix 11 – Newcastle Ottawa Scale for Case-Control and Case-Test Negative Studies

| **Author, year** | **Representativeness of the exposed cases** | **Selection of the non-exposed cases** | **Exposure** | | **Case Definition** | **Definition for controls** | **Comparability** | |
| --- | --- | --- | --- | --- | --- | --- | --- | --- |
|  |  |  | **Ascertainment of exposure** | **Same method of ascertainment for cases and controls** |  |  | **Comparability of cases on the basis of the design or analysis** | **Reporting of non-response rate** |
| Shah, 2020 | No Star | Star | Star | Star | Star | Star | Star | No Star |
